# Organ‐Specific Human Microbiomes and Dysbiosis: Mechanistic Links to Disease and Emerging Therapeutic Strategies

**DOI:** 10.1002/jcla.70307

**Published:** 2026-07-06

**Authors:** Awadh Alanazi

**Affiliations:** ^1^ Department of Clinical Laboratory Sciences, College of Applied Medical Sciences Jouf University Sakaka Saudi Arabia

**Keywords:** dysbiosis, gut microbiota, gut‐organ axis, microbial colonization, oral microbiome

## Abstract

**Background:**

The human microbiome is a dynamic and diverse community of microorganisms that affects susceptibility to illness and promotes wellness. Dysbiosis, or disruption of this delicately regulated microbial ecology, has been identified as a major factor in the emergence and development of systemic and organ‐specific disorders.

**Objective:**

With an emphasis on dysbiosis‐driven illness processes and therapeutic intervention implications, this study attempts to critically analyze host–microbiome interactions across key human organ systems.

**Methods:**

Using predetermined microbiome‐related keywords, a systematic literature search (2001–2025) was carried out in PubMed, Scopus, Web of Science, and Google Scholar. To assess microbiome formation, organ‐specific distribution, disease correlations, and therapeutic implications, English‐language peer‐reviewed original papers, meta‐analyses, and clinical or validated animal studies were chosen and methodically compiled.

**Results:**

Microbiome dysbiosis is linked to cardiovascular, metabolic, inflammatory, neurological, hepatic, renal, and cancer‐related illnesses by interfering with immune modulation, metabolic balance, and epithelial barrier integrity, according to evidence from human and verified animal research. Modified production of short‐chain fatty acids, immunological signaling imbalance, chronic inflammation, and communication between the gut–organ axis are examples of mechanistic linkages. Immune and metabolic indicators improved condition‐specifically with interventions such as probiotics, fecal microbiota transplantation, and diet‐based regulation.

**Conclusion:**

Collectively, current evidence supports the microbiome as a modifiable determinant of disease risk and therapeutic response, underscoring its translational potential for precision medicine.

## Graphical abstract

Schematic overview of the human microbiome and major microbiota‐derived metabolites across body sites, highlighting the gut–brain, gut–heart, and gut–kidney axes in host physiology and disease.

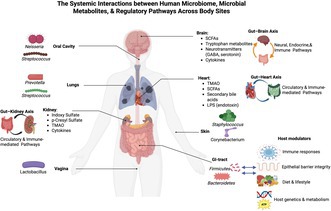

## 1 Introduction

A host and its microbiome together constitute a holobiont, and their genes are the hologenome [1]. The human microbiome is made up of different microbial communities called microbiota, which include bacteria, archaea, fungi, viruses, and other components. Microbiota colonize the skin, gut, mucosa, and, in some cases, intracellular environments. Through coevolution with the host, they may promote the maintenance of host health [2, 3]. The microbiome encompasses both these organisms and their genomes [3], including microorganisms, their components, metabolites, and host molecules that contribute to the shaping of their environment. Sometimes described as the “second genome,” the human microbiome contains between 50 and 100 times more genes than the human genome and extends the functions of the host through enzymatic and metabolic outputs that play a role in immunity, metabolism, and overall wellness [4].

The relationship between the host and the microbiome is critical to health because dysbiosis, the disruption of the normal balance of microbial communities within the body, especially the gut, is associated with a wide range of diseases. The most common characteristics of dysbiosis are a reduction in microbial diversity, a loss of beneficial microbiota, and an overgrowth of harmful microbiota [5]. True pathogens comprise a minority of microorganisms. While most microorganisms contribute to ecosystem stability and beneficial interactions with hosts, dysbiosis can facilitate the emergence of pathogens [6, 7].

Microbial composition differs between sites and individuals; however, probiotics, prebiotics, and synbiotics may be used to maintain the microbial balance. Determination of the core microbiota, that is, microbes consistently associated with either a host or an environment, enables differentiation between stable and transient species, thereby improving microbiome research [8]. Although the term microbiome is relatively recent, microbes have existed for over 4.5 billion years and have contributed to the evolution of humans. Microorganisms were first observed in 1675 and were later associated with diseases by scientists such as Pasteur and Koch. The beneficial roles of microbes in symbiosis, soil health, and fermentation were recognized even though the 20th century was dominated by antibiotics. A breakthrough discovery in the field came in the 1960s when Carl Woese classified bacteria by using 16S rRNA genes to establish a molecular phylogeny. This technique opened up a new field of metagenomics, and DNA sequencing of microbial communities could be performed directly to reveal the presence of a diversity of uncultivated microbes with their numerous functions [3].

An overview of the human microbiome, its function in health and illness, and treatment interventions is provided in this article. Mechanistic insights, inter‐individual variability, and translational obstacles are still poorly understood despite the connections between dysbiosis and chronic disorders. This review highlights important interventions, population variability, and reproducibility limits to guide personalized microbiome‐based diagnostics and treatments by incorporating recent human and verified animal investigations.

## 2 Review Methodology

This review undertook a meticulous and methodical search of peer‐reviewed literature on the human microbiome. It employed the key terms “human microbiome,” “dysbiosis,” “gut–organ axis,” “microbiome‐related diseases,” and “microbiome therapeutics” to search key scientific databases such as PubMed, Scopus, Web of Science, and Google Scholar. Only English language articles were reviewed. Peer‐reviewed original studies, reviews with meta‐analyses, and clinical studies that used human subjects or verified animal models published from 2001 to 2025 were included. The articles were evaluated according to predefined review criteria and then synthesized to discuss the role of the microbiome in terms of its development, distribution in the human body, relationship to the immune system, disease association, and therapeutic possibilities.

## 3 Factors Influencing the Human Microbiome

All parts of the body, including the skin, eyes, nails, respiratory, renal, urogenital, and reproductive systems, have microbes. Instead of colonizing random surfaces, microbes select areas that suit their preferences, which are influenced by a variety of factors such as temperature, pH, oxygen level, salt concentration, pressure, and nutrient availability. For example, sebum, which is rich in carbon and changes the pH, is vital nutrient source for bacteria [9] and the mucus of the digestive tract provides nutrients for microorganisms [10].

Both internal and external factors of the host affect the microbiome. Body site features, genetics, age, gender, and ethnicity are examples of internal influences. Environmental exposures, drugs, diet, and lifestyle are examples of external variables. Furthermore, the early years are crucial because the way a child is delivered affects the development of the early gut microbial communities, which stabilizes after approximately 3 years of age [11]. Later in life, decreased immunity and slower digestion typically lead to a decrease in beneficial microorganisms such as *Bifidobacterium*, causing dysbiosis [12]. Microbial communities can flourish in stable environments, but they can also be disrupted by environmental changes, which may lead to disease. Factors influencing the human microbiome are summarized in Table 1.

**TABLE 1:** Factors influencing the human microbiome.

Factor	Characteristics	Effect on microbiome composition/Function	References
Body Site	Skin, gut, lungs, urogenital system, reproductive tract	Microbes settle where conditions are favorable (temperature, pH, oxygen, salt levels, pressure, nutrients, etc.)	[9, 10]
Genetics	Host genes, sex, ethnicity	Influence baseline microbial diversity and composition	[11]
Age/Development	Infancy, childhood, adulthood, older age	Early‐life colonization sets initial gut microbiota (the mode of delivery is important); stabilizes ~3 years of age; aging reduces beneficial microbes like *Bifidobacterium* spp.	[12]
Diet	High‐fiber, high‐fat, vegan, breastfeeding	Alters microbial diversity and metabolite production	[11]
Lifestyle/Environment	Exercise, stress, urban vs. rural, pollution	Modulates microbial composition via immune and metabolic pathways	[11]
Medications	Antibiotics, proton pump inhibitors, nonsteroidal anti‐inflammatory drugs	Can disrupt microbial balance, reduce diversity, and promote dysbiosis	[12]
Local secretions/Nutrients	Sebum, mucus	Provides nutrients and attachment sites for microbes; shapes site‐specific communities	[9, 10]

## 4 Human Microbial Development, Personalization, and Stabilization

Beginning at birth, the microbiome undergoes rapid primary succession until stabilizing into a comparatively stable climax community in adolescence [13]. Although generally stable, adult microbiomes can fluctuate, and perturbations like antibiotics or infections can cause secondary succession that can shift the ecosystem into a new microbial state [14, 15]. In old age, microbial diversity tends to decline, marking the final phase of succession [15].

The microbial composition of the human microbiome is highly individualized and shaped by both host and environmental factors, but the microbial communities tend to be resilient and stable over time [16, 17]. Longitudinal studies such as iHMP have shown that the fecal and oral microbiota are relatively more stable over time compared to skin and nasal passage microbiota. Microbiota with highly individualized signatures are more reliably controlled by the host [18]. Some microbial communities change predictably with human development and, in some cases, can be used to accurately estimate chronological age, with the skin microbiota showing greater accuracy (mean error ± SD: 3.8 ± 0.45 years) than the oral and gut microbiota [18, 19].

## 5 Human Microbial Distribution

**FIGURE 1:**
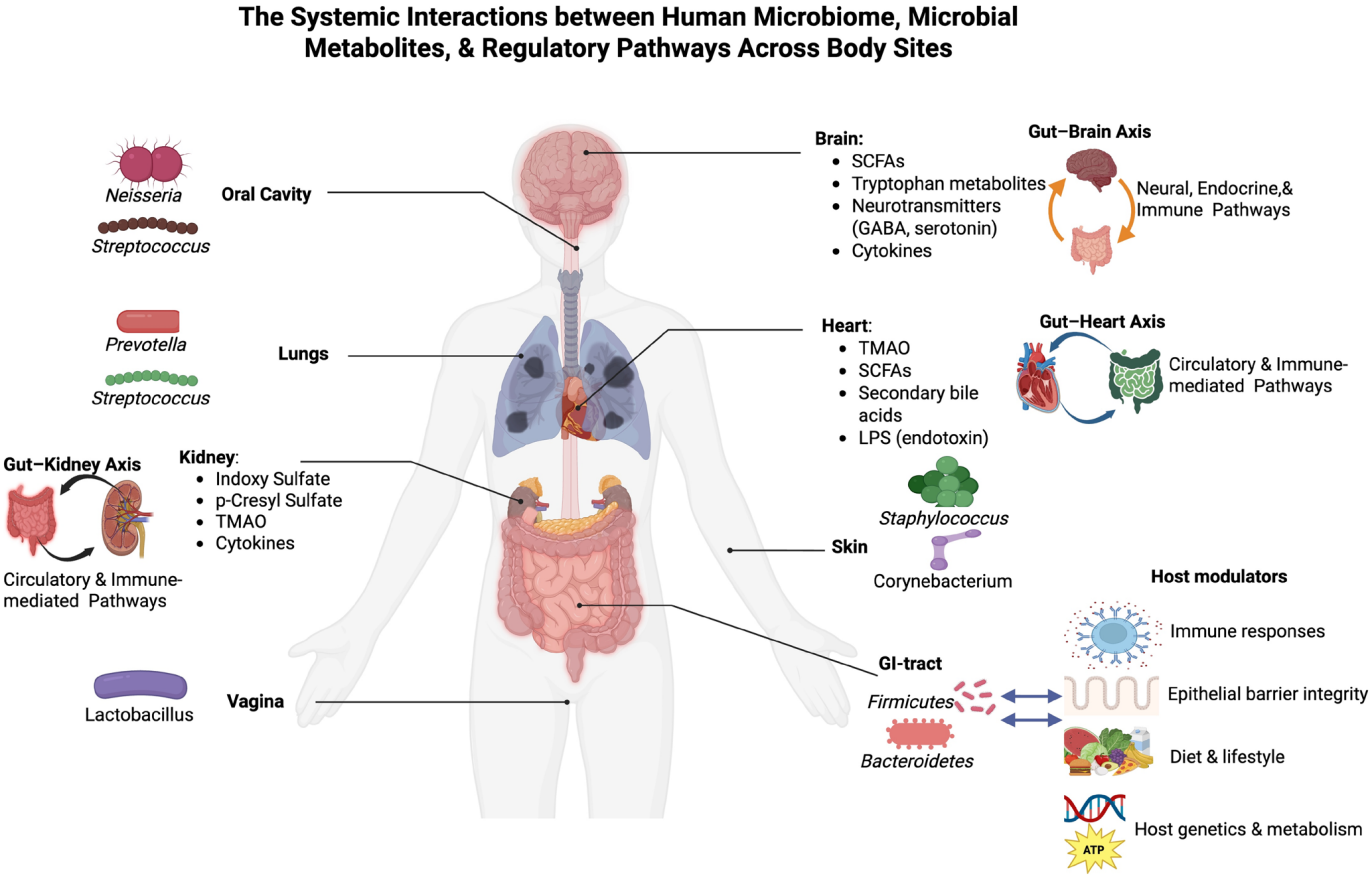
Systemic interactions between the human microbiome, microbial metabolites, and host regulatory pathways across body sites. Major microbial communities and microbiota‐derived metabolites (e.g., short‐chain fatty acids [SCFAs], trimethylamine N‐oxide [TMAO], tryptophan metabolites, neurotransmitters, lipopolysaccharide [LPS], indoxyl sulfate, and p‐cresyl sulfate) in different body sites are shown, highlighting the gut–brain, gut–heart, and gut–kidney axes involved in host regulation and disease. Metabolite signaling occurs through immune (cytokines, LPS), neural (vagus nerve, neurotransmitters), and endocrine–metabolic (circulatory and bile acid–mediated) pathways. Bidirectional interactions are impacted by host characteristics like immune state, the integrity of the epithelial barrier, genetics, and environmental exposures; routes are shown graphically to highlight key ideas rather than provide comprehensive mechanisms.

The human body provides a habitat for numerous microorganisms such as bacteria, fungi, archaea, and bacteriophages (Figure 1). These microorgabisms have co‐evolved with humans, and their populations change through each stage of life [15, 19]. They occupy the mucosal and superficial layers of organs and continually interact with their surroundings. The gut is heavily colonized by bacteria, with approximately 29% of the total microbial community, followed by the oral cavity (about 26%), skin (about 21%), and respiratory and urogenital tracts (about 14% and 9%, respectively). Moreover, the density of microbes varies within an organ; there are more microbes in the upper parts of the respiratory tract or the distal parts of the intestines than in their lower or proximal parts [20].

## 6 Microbiomes of Human Body Sites

### 6.1 The Digestive Tract Microbiome

Colonization of the gut begins at birth, with babies picking up pioneering microbes from the mother through the vaginal canal, by skin contact, and likely from fecal exposure at the time of delivery. The first colonizers of the gut are primarily *Bifidobacterium* spp., but over the first year, the community expands to a diverse group of *Bifidobacterium*, *Clostridium*, and *Bacteroides* spp. [21]. This transition yields greater Firmicutes diversity, including the introduction of *Clostridium*, *Faecalibacterium*, *Ruminococcus*, and *Veillonella*, with *Bifidobacterium* spp. generally declining. By 3 years of age, the gut microbiota reach a more stable configuration, with Firmicutes and Bacteroidetes dominating [22].

Most studies depend on stool samples, which may not reflect actual intestinal diversity [23]. It is now possible to show where particular microorganisms live along the gut using methods like ingestible capsules and postmortem investigations. For example, *Helicobacter* lives in the stomach and esophagus, *Prevotella* in the duodenum, *Enterococcus* and *Bacteroides* in the ileum, and a combination of *Klebsiella*, *Enterococcus*, *Lactobacillus*, *Parabacteroides*, *Bifidobacterium*, and *Dorea* in the colon. These patterns are linked to bile acid management, amino acid synthesis, fermentation, short‐chain fatty acid (SCFA) synthesis, and gut motility regulation [24, 25]. On the whole, diversity tends to be lower in the esophagus and stomach than in the small intestine and colon and is starkly different between mucosal surfaces and the lumen, underlining the spatial complexity of the gut microbiome [25, 26].

### 6.2 The Oral Microbiome

The oral cavity hosts a bacterial community from birth, which includes *Streptococcus*, *Gemella*, *Granulicatella*, and *Veillonella* populations. In early life, *Lactobacillus* and *Fusobacterium* start to increase [27]. By approximately 3 months of age, *Staphylococcus* peaks and then gradually declines, allowing the growth of *Gemella*, *Granulicatella*, *Haemophilus*, and *Rothia* spp. [27]. With the eruption of teeth, the microbial community changes once again, with higher levels of Fusobacteriota, Synergistetes, Tenericutes, Saccharibacteria (TM7), and SR1 by adulthood [28, 29]. In a study of over 2000 saliva samples, 68 core taxa were identified. Of these, *Streptococcus oralis* subsp. *dentisani* is highly beneficial for oral health, *Neisseria* is one of the dominant populations in saliva and is linked to lipid metabolism, and *Lautropia*, *Veillonella*, and *Atopobium* are other key players contributing to metabolic functions [30].

### 6.3 The Skin Microbiome

At birth, the newborn's skin is first populated mainly by the mother's vaginal *Lactobacillus* spp., but within 4 and 5 weeks, the microbiota shift toward a pattern closer to that of adult skin [31]. As adolescence arrives, the community becomes more specialized by body site. The core players are *Staphylococcus* and *Corynebacterium*, with other genera like *Pseudomonas, Enterobacter*, *Enterococcus*, *Proteus*, and *Klebsiella* occurring more in specific areas such as the armpit and forearm [32]. Regional variations also exist. There is a preponderance of *Propionibacterium acnes* in oily regions, whereas *Corynebacterium* and *Staphylococcus epidermidis* are abundant in arid regions and an overgrowth of the fungi *Malassezia globosa* and *Malassezia restricta* is present in moist regions. A unique set of microbes is found under toenails. The skin microbiome serves as a reservoir for antibiotic resistance genes, which vary between individuals and between body sites [33].

### 6.4 The Vaginal Microbiome

The vagina is generally dominated by a single *Lactobacillus* species. Before puberty, low glycogen and estrogen levels support a diverse flora, consisting of streptococci, enterococci, and several anaerobes [34]. During the reproductive stage, higher levels of estrogen promote the predominance of one of four *Lactobacillus* species: *L. crispatus* , *L. iners* , *L. jensenii* , or *L. gasseri* [35]. In contrast, menopause suppresses lactobacilli and increases the diversity of the anaerobic community; this transition is associated with an increased risk of bacterial vaginosis [36]. However, about 25% of North American women are colonized by a diverse microbial community, featuring both anaerobic and aerobic taxa, including *Gardnerella*, *Prevotella*, and *Atopobium*, rather than lactobacilli [37]. Comparison of communities across ethnicities showed that the most common members are *Lactobacillus iners* , *Lactobacillus crispatus* , and *Gardnerella vaginalis* [38].

### 6.5 The Respiratory Microbiome

The upper airway harbors distinct microbial communities; the nasal cavity and nasopharynx are rich in *Moraxella*, *Staphylococcus*, *Corynebacterium*, *Haemophilus*, and *Streptococcus* spp., whereas the oropharynx tends to harbor *Prevotella*, *Veillonella*, *Streptococcus*, *Leptotrichia*, *Rothia*, *Neisseria*, and *Haemophilus* spp. [39, 40]. Depending on infant delivery mode, either *Ureaplasma* or *Staphylococcus* predominates in the lower respiratory tract. *Staphylococcus* dominates after cesarean delivery and *Ureaplasma* after vaginal delivery, but by the second month, this community expands to include oral commensals, such as *Streptococcus*, *Prevotella*, *Porphyromonas*, and *Veillonella* [41]. In general, the lower respiratory tract maintains a low microbial biomass due to immune defenses and clearance mechanisms for efficient gas exchange [42].

## 7 The Role of the Microbiome in Immune System Development

Microbial colonization of mucosal surfaces in early life is crucial for the maturation of the immune system [43]. The critical window for microbiota development is in the first 3 years of life, when infants are more susceptible to environmental perturbations that can have long‐lasting immune consequences [44]. Immature immunity predisposes newborns to infections, and remains one of the most common causes of mortality in childhood [45]. Preterm infants face additional risks due to excessive inflammation, including necrotizing enterocolitis [46].

Evidence suggests that substantial microbial colonization does not occur in utero [47]. The primary seeding following birth originates from the mother's microbiota [13]. The early composition of the microbiome is thus substantially influenced by the mode of delivery of the infant. Breastmilk provides passive immunity via personalized maternal antibodies [48]. The maternal microbiota may also extend antibody‐mediated protection to infants during breastfeeding [49].

Germ‐free (GF) models illustrate how microbiota drive immune development. In the absence of microbes, GF animals exhibit intestinal immune defects, including fewer intraepithelial lymphocytes and low IgA levels, both of which rebound on colonization. Maternal colonization during pregnancy confers increased ILC3s and other mononuclear cells on the offspring. Th17 cells, abundant in the small intestine, are absent in GF mice but can be reconstituted through colonization, particularly by segmented filamentous bacteria (SFB) and other commensals. This colonization appears to be associated with SFB attachment to epithelial cells. *Bacteroides fragilis* polysaccharides also promote immune maturation, correcting T‐cell balance and Th1/Th2 skewing. Early in life, microbiota guide B cell development and antibody repertoires and contribute to regulatory networks that limit mucosal IgE responses associated with allergy risk [50]. Neonatal recognition of flagellin via Toll‐like receptor 5 (TLR5) also dictates which flagellated bacteria persist and determine the long‐term immune balance [51]. Thus, early life represents a critical window during which microbiota and immune interactions establish lasting vulnerability or resilience to infection and inflammation.

## 8 Colonization Resistance Against Pathogens

Microbial communities residing in the gut, skin and, on mucosal surfaces play a key role in protecting against diseases through colonization resistance as first defined by Bohnhoff et al. [38]. These resident microbes restrict invading organisms through competition for limited nutrients, production of antimicrobial compounds, and modulation of bacteriophage activity [52].

In the gut, non‐pathogenic *Escherichia coli* regulates the major foodborne pathogen *E. coli* O157:H7 through competition for nutrients, while *Bacteroides* species assist in polysaccharide breakdown and suppress the opportunistic *Enterobacteriaceae* pathogens [53]. Freter's niche hypothesis proposes that the microbial community is determined by substrate availability [38]. This concept is supported by mouse experiments, as knocking out a single sugar source affects the entire microbiota and alters susceptibility to infections [54]. Additionally, the gut microbiota suppresses pathogenic competitors using bacteriocins and secretion systems Type VI and Type VII [55, 56]. Bacteriophages further regulate microbial populations, with bacteria deploying defense strategies such as the CRISPR‐Cas system and abortive infection mechanisms [57, 58].

The vaginal microbiome supports the host defense system through colonization resistance and is organized into five main community states dominated by *Lactobacillus* species. Lactobacilli protect against sexually transmitted and urinary tract infections by producing lactic acid and bacteriocins [38]. Decreases in the number of *Lactobacillus* species increase susceptibility to HIV, HPV, HSV, and bacterial vaginosis infection [38]. Additionally, the balanced microbial makeup of the skin prevents the growth of pathogens, while dysbiosis is linked to skin conditions, such as infection by *Propionibacterium acnes*. Furthermore, the skin microbiota produces antibacterial agents, such as those produced by *Staphylococcus epidermidis* , which inhibit *Staphylococcus aureus* biofilm formation, and the compound lugdunin, an antibiotic produced by *Staphylococcus lugdunensis* , which prevents the growth of various pathogenic bacteria [38].

## 9 The Microbiome in Disease Development

**FIGURE 2:**
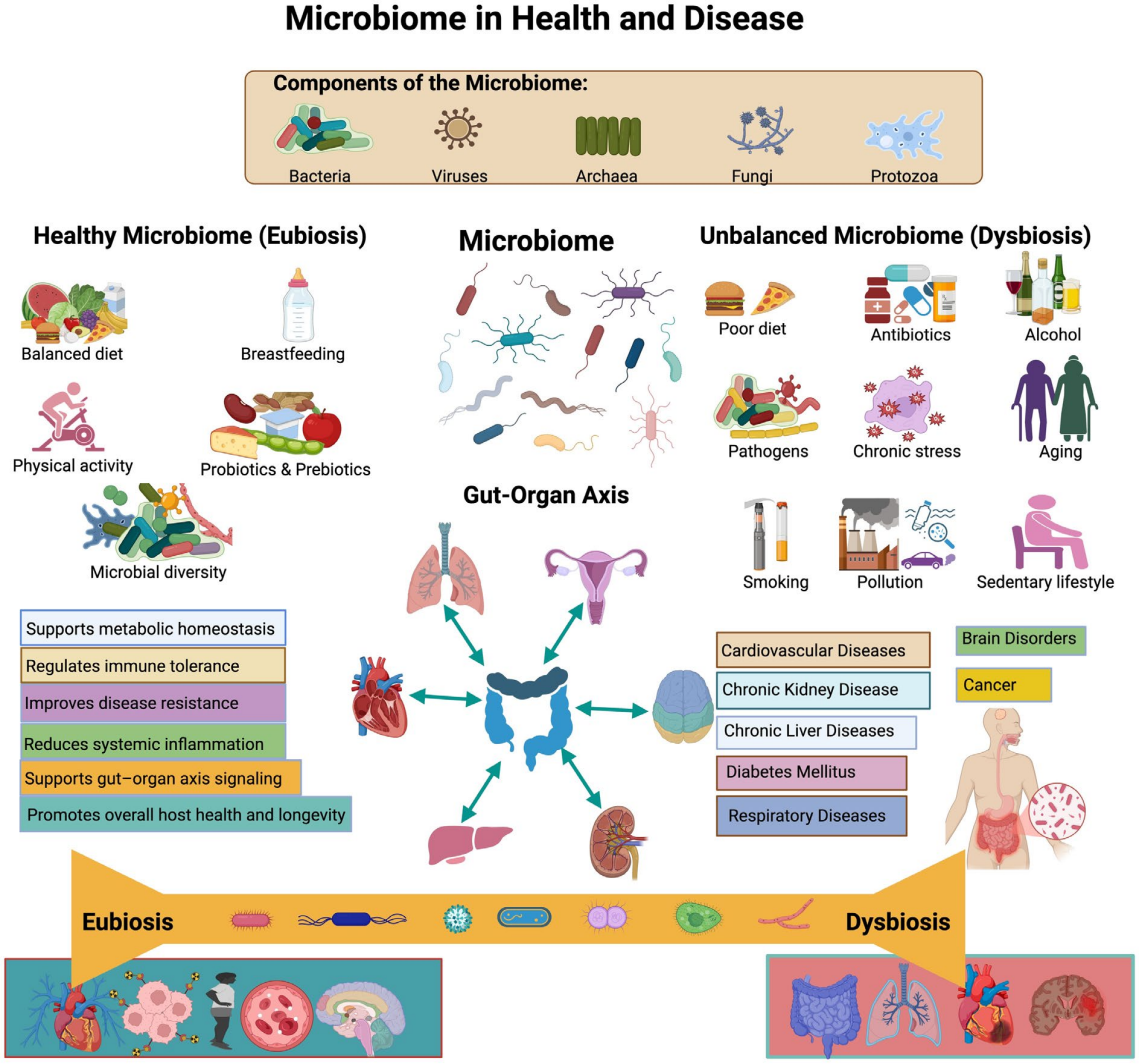
The human microbiome as a central regulator of health and disease. A balanced microbiome (eubiosis) is contrasted with dysbiosis to highlight key microbial components and lifestyle and environmental factors determining microbial balance, gut–organ axis interactions, and major disease outcomes associated with microbiome disruption. Importantly, the majority of the associations between microbiomes and diseases that are shown are associative correlations; causative contributions have only been proven in certain situations (e.g., certain infections and toxin‐mediated illnesses).

The gut's microbial community represents a complex network of trillions of microbes. Newer technologies in sequencing are beginning to illustrate how changes in this microbial composition are related to disease. External factors can offset the balance, promoting dysbiosis and functional alterations that provide a context for various diseases (Figure 2), including cardiovascular diseases (CVDs), cancer, respiratory and metabolic disorders, inflammatory bowel disease (IBD), neurological disorders, and liver and kidney disease [59].

### 9.1 Cardiovascular Diseases

Atherosclerosis, high blood pressure, obesity, diabetes, and abnormal lipid levels are known risk factors of CVDs, which continue to be the world's leading cause of disease and death [60]. According to recent research, dysbiosis causes the start of disease, whereas microbiota are linked to cardiovascular health. Microbial transplantation, metabolic pathway, and microbial metabolite studies suggest that microbial communities can modify food metabolism and affect cardiovascular functions [61].

Periodontal disease, which is caused by dysbiosis of the oral flora, is linked to a higher risk of CVD. For example, DeStefano and colleagues found that people with periodontitis had a roughly 25% higher risk of CVD, and this link was further supported by the presence of common oral‐plaque bacterial taxa in atherosclerotic plaques [62]. Two potential mechanisms were proposed by Schenkein and colleagues: (1) inflammatory mediators such as C‐reactive protein (CRP), fibrinogen, and metalloproteinases may leak into the bloodstream; and (2) oral pathogens may infiltrate endothelial cells or phagocytes and cause lesion formation. In a previous study, the antibody levels against *Tannerella forsythia* was inversely associated with mortality due to CVD [63], whereas other studies linked periodontitis to major cardiovascular risk factors. Indeed, intensive periodontal treatment has been found to reduce IL‐6, CRP, and systolic blood pressure and improve lipid profiles [64]. In patients with periodontitis, elevated levels of the markers E‐selectin, myeloperoxidase, and ICAM‐1 indicate endothelial activation and an increased risk of CVD [65].

### 9.2 Cancer

Cancer comprises a diverse set of diseases arising from genetic and epigenetic alterations that drive uncontrolled cell proliferation and dissemination [66, 67]. Healthy lungs have diverse bacterial assemblages, with genera such as *Pseudomonas*, *Streptococcus*, *Prevotella*, and *Veillonella*. In contrast, patients with lung cancer show increased abundance of *Actinomyces*, *Veillonella*, *Streptococcus*, *Megasphaera*, and *Mycobacterium*, with some *Enterobacteriaceae* taxa associated with poorer survival [68, 69]. Greater microbial diversity in adjacent normal tissue may indicate poor prognosis. Lung dysbiosis can promote cancer by enhancing inflammation, stimulating γδ T cells [70], and activating oncogenic PI3K/ERK pathways [71].

In the gut, dysbiosis promotes colorectal cancer (CRC) by weakening the mucosal barrier and promoting inflammation [72] via several pathways, including E‐cadherin/β‐catenin, Toll‐like receptor 4 (TLR4)/MYD88/NF‐κB, and SMO/RAS/p38 MAPK pathways [73]. Commensal and opportunistic microbes take advantage of the defects in the gut mucosal barrier to invade into tissue and generate genotoxins [74]. Studies indicated notable bacteria associated with CRC include *Enterococcus faecalis* , *Escherichia coli* , *Bacteroides fragilis* , *Streptococcus bovis* , *Fusobacterium nucleatum* , and *Helicobacter pylori* [75], among which *F. nucleatum* is frequently enriched in CRC tissue and has been shown to invade tumor cells [76].

### 9.3 Diabetes Mellitus

Diabetes mellitus includes type 1 diabetes mellitus (T1DM), type 2 diabetes mellitus (T2DM), and gestational diabetes mellitus (GDM). All types of diabetes are marked by impaired blood glucose regulation. Increasing evidence associates microbiota imbalance with the initiation and development of each type of diabetes mellitus [62].

#### 9.3.1 Type 1 Diabetes Mellitus

In T1DM, both gut and oral microbial communities show signs of dysbiosis. Most notably, fecal samples often have a greater abundance of *Christensenella* and *Bifidobacteria*. The oral cavity has higher levels of *Streptococcus* and the gut has fewer streptococci and shows a reduction in SCFA‐producing bacteria like *Ruminococcus faecis* , *Faecalibacterium prausnitzii* , and *Intestinimonas* [77, 78]. Higher levels of IL‐1β, IL‐6, and TNF‐α and lower levels of IL‐10 and IL‐13 are indicative of the persistent inflammation of the pancreatic islets caused by this dysbiosis. Lipopolysaccharide (LPS) signals and the particular microbial array both affect the inflammatory signature [77, 79]. As evidenced by the protection observed in MyD88‐deficient non‐obese diabetic mice, innate immune mechanisms also contribute through signaling by TLR2/4 and the MyD88/NF‐κB axis [80, 81].

#### 9.3.2 Type 2 Diabetes Mellitus

The gut microbiota in T2DM is marked by specific changes, such as a decrease in the genera that produce SCFAs, including *Faecalibacterium* and *Roseburia*, a high Bacteroidetes/Firmicutes ratio, and reduced typical Firmicutes and Clostridia [82, 83]. This dysbiosis leads to chronic inflammation, and a high LPS level increases TLR4/NF‐κB signaling, potentially impacting insulin secretion. Altered bile metabolism and reduced SCFA availability further disrupt glucose regulation, intestinal barrier function, and regulatory T cell development [84, 85]. Additionally, studies have shown that changes in the oral microbiome and a high IL‐17 level may also impact gut immunity and link T2DM and periodontitis [86, 87].

#### 9.3.3 Gestational Diabetes Mellitus

Similar microbial changes occur during pregnancy in GDM. Changes in gut microbiota contribute to the development of insulin resistance and inflammation during pregnancy [88]. Microbiota diversity generally increases, but species richness decreases from early to late pregnancy [89]. GDM is associated with an increased *Firmicutes/Bacteroidetes* ratio and reduced numbers of SCFA producers that favor hyperglycemia [90, 91]. Specific taxa are associated with metabolic signaling pathways, including *Collinsella* for insulin signaling, *Coprococcus* for gut‐peptide signaling, and *Ruminococcaceae*/*Lachnospiraceae* for adipokines [92]. This imbalance of the maternal microbiome does not remain stationary but affect the infant. Thus, infants have more pro‐inflammatory bacteria, reduced alpha‐diversity, and shifted lactic acid bacteria, indicating early‐life microbiota impacts [93, 94].

### 9.4 Respiratory Diseases

Respiratory ailments range from chronic conditions, such as asthma and chronic obstructive pulmonary disease (COPD), to scarring of the lungs, known as pulmonary fibrosis, and include infectious diseases like pneumonia. A growing body of evidence has shown that microbes in the mouth, lungs, and gut play a meaningful role in how these diseases originate and progress [62].

#### 9.4.1 Chronic Respiratory Diseases

Asthma and COPD have different lung microbial profiles. Asthma patients tend to harbor more Proteobacteria, while COPD exacerbation involve fungi and bacteria such as *Pseudomonas*, *Moraxella*, *Lactobacillus*, and *Haemophilus*. Pulmonary fibrosis is associated with higher levels of *Staphylococcus* and *Streptococcus* [62]. Microbes can drive inflammation; for example, Proteobacteria drive Th17/IL‐17 responses, *Haemophilus influenzae* can cause steroid resistance, and *Moraxella* can exacerbate allergic inflammation [95]. The gut–lung axis is also important. Early life disruptions increase the risk of asthma [96]; gastrointestinal diseases are associated with asthma and COPD; and COPD exacerbations are associated with increased gut permeability and higher trimethylamine N‐oxide (TMAO) [97, 98]. Studies show that lower levels of *Veillonella*, *Faecalibacterium*, and *Lachnospira* in early life increase the risk of asthma [99] and SCFAs promote regulatory T cells and suppress airway inflammation [100]. Oral bacteria also play a key role in the lungs, with *Veillonella*, *Prevotella*, *Fusobacterium*, and *Porphyromonas* being associated with airway inflammation [101, 102]. Inflammatory biomarkers, including TNF‐α and CRP, are elevated in asthma and COPD [62].

#### 9.4.2 Pneumonia

Lung or gut microbiota dysbiosis increases the risk of pneumonia. Children with low nasal diversity is dominated by *Lactobacillus*, *Rothia*, or *Streptococcus*, or showing early colonization by *Streptococcus pneumoniae* , *Haemophilus influenzae* , or *Moraxella catarrhalis* are particularly vulnerable, as are HIV patients with higher levels of *Prevotella* and *Veillonella* [62]. Disrupted SCFAs suppress the responses of IFN‐γ and IL‐17A [103], whereas lung‐tolerant microbiota facilitate influenza‐specific IgA [104]. Dysbiosis is present in COVID‐19 pneumonia, which is characterized by an increase in *Acinetobacter* and *Cryptococcus*, while a shift toward increased *Klebsiella*, *Faecalibacterium*, and *Rothia* is also seen [105, 106]. The gut microbiota also influence disease severity: depletion tends to worsen the course of *Streptococcus pneumoniae* infection, while segmented filamentous bacteria confer protection, and severe COVID‐19 is associated with the loss of commensal organisms and high levels of inflammation [107, 108].

### 9.5 Inflammatory Bowel Disease

Inflammatory bowel disease (IBD), which includes Crohn's disease and ulcerative colitis, arises from a complex interplay between immune dysregulation, barrier dysfunction, genetic susceptibility, and environmental influences [109]. Gut dysbiosis accelerates the course of disease by promoting mucosal invasion and permitting pathogenic *Escherichia coli* to proliferate [62], whereas commensals are reduced and Proteobacteria taxa are increased [110]. Although evidence of causality is still pending, enterohepatic *Helicobacter* species may also play a role [111]. New data implicate an oral–gut axis through which oral pathobionts can migrate into the gut and promote inflammation [112]. Periodontitis has been associated with IBD, with changes in salivary microbiota are associated with cytokine levels [113] and Crohn's disease exhibiting increased periodontal markers [114].

### 9.6 Brain Disorders

Brain tumors are complicated by aberrant protein expression and the development of drug resistance [115]. Microbial dysbiosis contributes to neuropsychiatric and neurodegenerative diseases through inflammation, gut–brain axis signaling, and immune modulation. Depression is associated with an increased risk of death [116] and microbiota‐driven hyperactivation of the hypothalamic–pituitary–adrenal (HPA) axis [117].

#### 9.6.1 Neuropsychiatric Disorders

The gut–brain axis plays a key role in modulating behavior, with animal studies demonstrating impacts on cognition and the stress response [118]. Stress‐induced intestinal permeability enables endotoxins to provoke systemic inflammation, which contributes to depression and anxiety [119]. Major depressive disorder is associated with changes in microbiota, including elevated levels of *Bacteroidetes*, *Proteobacteria*, and *Actinobacteria*, as well as reduced *Firmicutes* [120] and depleted SCFA‐producing families such as *Lachnospiraceae* and *Ruminococcaceae* [121]. The neurotransmission signaling pattern is changed in GF animals, which indicates the effect of microbiota on neural function [122]. Gut dysbiosis affects neurodevelopment in autism spectrum disorder [123]; changes in *Clostridium* and *Ruminococcus* have been documented, and fecal microbiota transplantation has shown early advantages [124].

#### 9.6.2 Neurodegenerative Disorders

Dysbiosis is linked to stroke, Parkinson's disease, and Alzheimer's disease [125]. Microbial diversity and neurodevelopment are affected by early‐life variables including cesarean delivery [126]. Notable trends for Parkinson's disease include decreases in *Blautia*, *Faecalibacterium*, and *Ruminococcus* and increases in *Escherichia*, *Shigella*, *Streptococcus*, and *Enterococcus* [127], as well as other changes such as elevated *Prevotella* and *Akkermansia* [128] that exacerbate intestinal inflammation [129]. In Alzheimer's disease, there is a drop in Firmicutes and Actinobacteria and a rise in Bacteroidetes and Proteobacteria [130] associated with changes in cytokines and amyloid [131]. Reduced microbial diversity is also associated with stroke, and fecal microbiota transplantation shows some promise as an aid to neurological recovery [132].

### 9.7 Oral Dysbiosis

The microbiota profiles of saliva are distinct in children with autism spectrum disorder [133]. The mouths of adults with depression harbor high levels of *Prevotella nigrescens* and *Neisseria* [134]. Smoking and drinking distort the mouth's microbiome balance [135]. An altered mix of oral bacteria has also been observed in Parkinson's disease [136] and Alzheimer's disease [137].

### 9.8 Chronic Kidney Disease

Chronic kidney disease affects approximately 9% of the global population [138]. Diabetes, high blood pressure, and heart conditions are major risk for CKD [139]. CKD is clinically diagnosed when the glomerular filtration rate falls below 60 mL/min/1.73 m^2^ or when albuminuria in the urine has lasted for 3 months or more, resulting in irreversible kidney damage. Gut dysbiosis is closely related to the acceleration of CKD [140], largely due to diet‐ and treatment‐related inflammation and uremic toxin accumulation [141]. CKD and end‐stage renal disease are marked by a decline in beneficial microbes and an overgrowth of harmful microbes [142, 143]. This imbalance promotes an increase in gut‐derived toxins such as indoxyl sulfate, p‐cresyl glucuronide, p‐cresyl sulfate, and TMAO [144], whose levels are significantly increased in CKD [145, 146] and cause renal damage by impairing renal transporters, thus causing tubular injury [147, 148]. These uremic toxins drive inflammation, fibrosis, cardiovascular risk, and high mortality [9]. This process is exacerbated by increased gut permeability and decreased production of protective metabolites [146]. This evidence suggests a vicious cycle in which oral and gut microbiota dysbiosis accelerates CKD through intertwined inflammatory, metabolic, and toxic pathways.

The gut–kidney axis connects the immune and metabolic processes of the gut with those of the kidneys [138, 149]. CKD often tends to deteriorate when both oral and gut microbiomes are disturbed. For instance, periodontitis accelerates systemic inflammation and is associated with worsening kidney function [150], indicated by the higher antibody levels against oral pathogens and the high levels of oral pathogens present in CKD [151]. Even in prospective studies, CKD patients with periodontitis show higher mortality rates [152].

### 9.9 Chronic Liver Disease

Chronic liver disease, primarily nonalcoholic fatty liver disease (NAFLD), nonalcoholic steatohepatitis (NASH), and alcoholic liver disease can progress to cirrhosis and liver cancer [153]. Cirrhosis is defined by extensive scarring, loss of the normal structure of the liver, and is a fatal terminal condition.

#### 9.9.1 The Gut–Liver Axis

The connection between the gut microbiota and the liver via bile acids and portal circulation is known as the “gut–liver axis.” Dysbiosis substantially contributes to the advancement of liver disease [154]. A loss of Bacteroidetes and changes in various microbial populations are linked to NASH and NAFLD, while there are elevated levels of Proteobacteria and Fusobacteria in cirrhosis [155, 156]. Changes in bile acid balance, intestinal hyperpermeability, and inflammation mediated by deoxycholic acid and NF‐κB activation are potential pathways [157]. Hence, the lipotoxicity associated with choline metabolism [158] and LPS/TLR4/TGF‐β signaling may further contribute to fibrosis [159]. The development of NAFLD and NASH is influenced by each of these mechanisms [160].

#### 9.9.2 Oral Microbiota in Liver Disease

Oral microbes and their byproducts can penetrate the bloodstream and contribute to liver disease. *Porphyromonas gingivalis* , associated with NAFLD/NASH in animal models [161], is found more frequently in NAFLD patients [162], indicating periodontitis as a possible risk factor [163]. Oral microbes and proinflammatory factors like LPS and peptidoglycan can migrate to the liver and evoke an immune response via Toll‐like receptors (TLRs) [164]. Cirrhosis patients usually have problems associated with oral health, including candidiasis and xerostomia [165]. In hepatitis B virus infections, oral bacterial taxa, including *Fusobacterium*, *Eubacterium*, and *Treponema*, can potentially affect gut microbiota and liver disease [166]. Table 2 summarizes changes in the microbiome associated with major diseases and their underlying mechanisms or the pathophysiological pathways involved.

**TABLE 2:** Diseases associated with microbial dysbiosis and their underlying pathophysiology.

Disease	Altered microbial taxa/Dysbiosis	Mechanism/Pathophysiology	References
Cardiovascular diseases	Oral dysbiosis, periodontal pathogens	Oral microbes invade endothelial cells, trigger inflammation (CRP, IL‐6), endothelial activation, and atherosclerosis	[62]
Cancer	Lung: ↑ *Actinomyces*, *Veillonella*, *Streptococcus*, *Megasphaera*, *Mycobacterium* Gut: ↑ *Enterococcus faecalis* , *Escherichia coli* , *Bacteroides fragilis* , *Streptococcus bovis* , *Fusobacterium nucleatum* , *Helicobacter pylori*	Promotes inflammation, immune evasion, tumor proliferation, genotoxin production, and activation of oncogenic pathways	[71, 73, 75]
Type 1 diabetes mellitus	Gut: ↑ *Christensenella*, *Bifidobacteria*, ↓ short‐chain fatty acid (SCFA) producers (*Ruminococcus*, *Faecalibacterium*, *Intestinimonas*) Oral: ↑ *Streptococcus*	Chronic pancreatic islet inflammation; elevated IL‐1β, IL‐6, and TNF‐α; reduced IL‐10/IL‐13; and lipopolysaccharide (LPS)‐mediated immune activation	[77, 78]
Type 2 diabetes mellitus	Gut: ↓ Firmicutes/Clostridia, ↑ Bacteroidetes/Firmicutes ratio, ↓ SCFA producers (*Faecalibacterium*, *Roseburia*) Oral: ↑ IL‐17‐associated bacteria	Chronic inflammation, LPS‐induced TLR4/NF‐κB activation, impaired insulin secretion, and dysregulated gut barrier and glucose tolerance	[62]
Gestational diabetes mellitus	Gut: ↑ Firmicutes/Bacteroidetes ratio, ↓ SCFA producers; microbial shifts affecting metabolic signals (*Collinsella*, *Coprococcus*, *Ruminococcaceae*, *Lachnospiraceae*)	Promotes insulin resistance and maternal inflammation; vertical transmission alters infant microbiota	[92]
Chronic respiratory diseases	Asthma: ↑ Proteobacteria COPD: ↑ *Pseudomonas*, *Moraxella*, *Lactobacillus*, *Haemophilus*; fungi Pulmonary fibrosis: ↑ *Staphylococcus*, *Streptococcus*	Inflammation via Th17/IL‐17, steroid resistance, and allergic inflammation; gut–lung axis affects early‐life asthma risk	[62]
Pneumonia	Low nasal diversity, ↑ *Lactobacillus*, *Rothia*, *Streptococcus*; early colonization by *Streptococcus pneumoniae* , *Haemophilus influenzae* , *Moraxella catarrhalis* ; ↑ *Prevotell*a, *Veillonella* in HIV; COVID‐19: ↑ *Acinetobacter, Cryptococcus*	Dysbiosis impairs SCFA‐mediated immunity, reduces IFN‐γ/IL‐17A responses, and worsens disease severity	[62]
Inflammatory bowel disease	↑ Proteobacteria, pathogenic *Escherichia coli* ; ↓ beneficial bacteria; oral pathobionts migrate to gut	Enhanced mucosal invasion, barrier dysfunction, inflammation, and oral–gut axis dysregulation; periodontitis linked to disease severity	[110, 112]
Neuropsychiatric disorders	↑ Bacteroidetes, Proteobacteria, Actinobacteria; ↓ Firmicutes, *Lachnospiraceae*, *Ruminococcaceae*	Gut–brain axis dysregulation; systemic inflammation; altered neurotransmission; anxiety, depression, and autism	[120, 121]
Neurodegenerative disorders	Parkinson's disease: ↓ *Blautia*, *Faecalibacterium*, *Ruminococcus*; ↑ *Escherichia*, *Shigella*, *Streptococcus*, *Enterococcus*, *Prevotella*, *Akkermansia* Alzheimer's disease: ↓ Firmicutes, Actinobacteria; ↑ Bacteroidetes, Proteobacteria	Neuroinflammation, altered cytokines, amyloid deposition, and stroke susceptibility; fecal microbiota transplantation shows potential	[127, 130]
Chronic kidney disease (CKD)	↑ Gut‐derived toxins: indoxyl sulfate, p‐cresyl sulfate, trimethylamine N‐oxide; ↓ beneficial bacteria	Gut dysbiosis promotes inflammation, fibrosis, renal damage; oral dysbiosis (periodontitis) exacerbates CKD; gut–kidney axis disruption	[62]
Chronic liver disease	↑ Proteobacteria, Fusobacteria; ↓ Bacteroidetes in NASH/NAFLD; oral microbes: *Porphyromonas gingivalis* , *Fusobacterium*, *Eubacterium*, *Treponema*	Dysbiosis disrupts the gut–liver axis and bile acid metabolism, increases gut permeability, inflammation, and fibrosis; oral microbes contribute via LPS/Toll‐like receptor signaling	[62]

## 10 Microbiome Therapeutics

**FIGURE 3:**
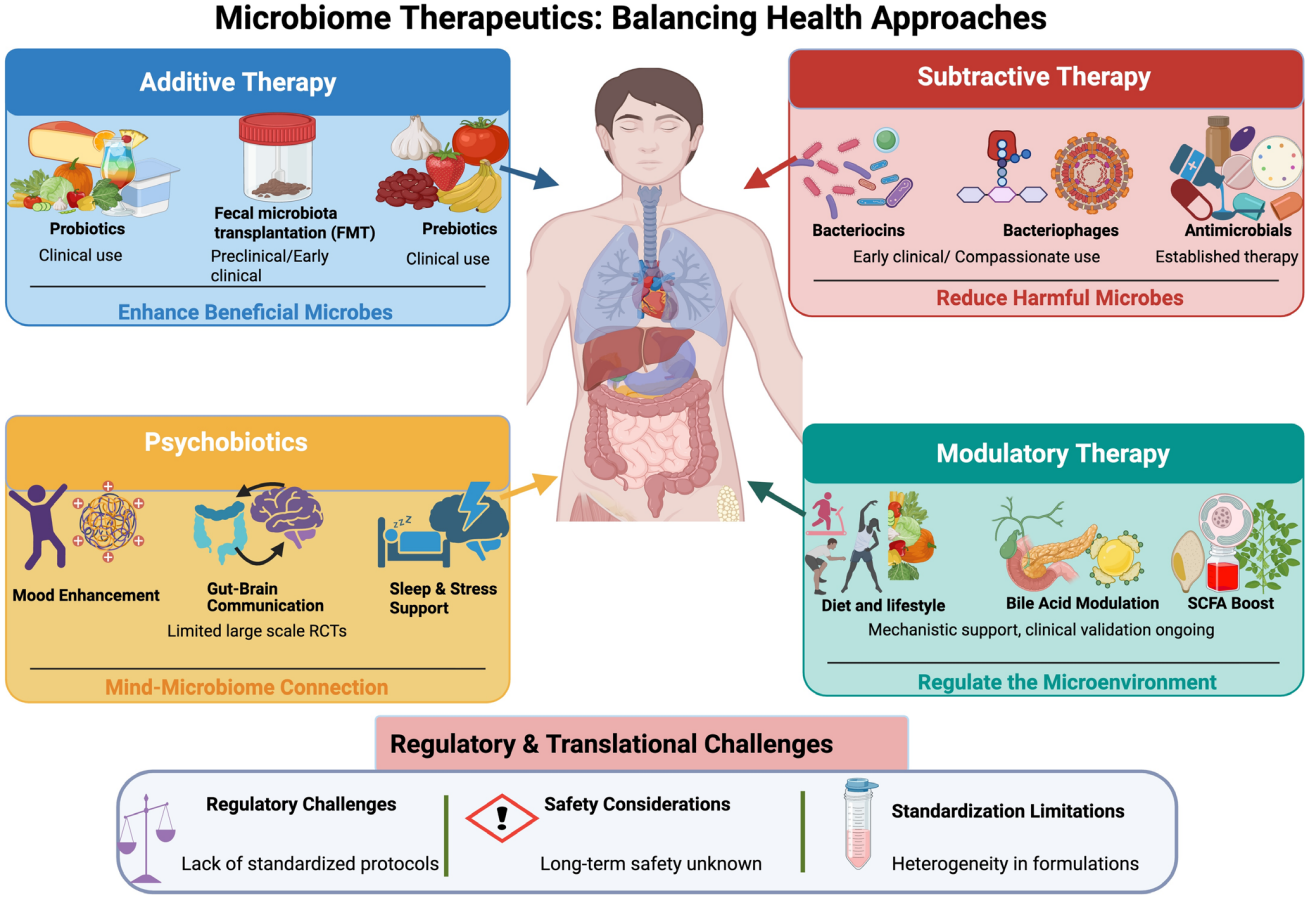
Microbiome‐based therapeutic strategies for restoring host health. Major microbiome‐targeted therapies, i.e., additive, subtractive, modulatory, and psychobiotic approaches, aimed at restoring microbial balance and supporting host physiological health are shown. Many modalities are still in early‐phase or preclinical stages, despite the fact that some (like FMT and antimicrobials) have entered clinical practice for specific indications. Widespread implementation is currently limited by regulatory frameworks, safety concerns, difficulties with standardization, and variations in host–microbiome responses.

Microbiome‐based therapies seek to modulate host–microbiome interactions using four major methods: additive, subtractive, modulatory, and psychobiotic [167]. Diet, prebiotics, bacterial communities, and microbial metabolites have emerged as strategies [168] for manipulating the microbiome by supplementing, depleting, or modifying host–microbiome interactions (Figure 3).

### 10.1 Additive Therapies

#### 10.1.1 Fecal Microbiota Transplantation

Fecal microbiota transplantation (FMT) shows restoration of gut microbial balance by transferring microbiota from healthy donors to sick recipients but faces greater safety, standardization, and regulatory challenges [169, 170]. It requires strict screening of fecal samples and blood testing. FMT uses dried stool transplants, filtrates, and bacterial cultures and demonstrates a very high effectiveness [171]. It is an efficient cure for recurrent *Clostridioides difficile* infection [172, 173, 174]. It is also effective against numerous metabolic disorders, infections, liver problems, and neurological disorders, and it acts as an adjuvant to cancer immunotherapy [175, 176, 177]. However, its efficacy shows variability across other disorders [178]. It is only a mildly effective treatment for ulcerative colitis and shows limited efficacy against Crohn's disease [179, 180]. The variation in efficacy is due to engraftment and genetic factors [181]. Safety issues, particularly among immunosuppressed patients, are driving the development of laboratory‐made therapies [182, 183].

#### 10.1.2 Probiotics

Probiotics are natural or engineered microorganisms, which, when administered in adequate amounts, show variable efficacy across the population. Common strains such as *Lactobacillus*, *Bifidobacteria*, and some *Escherichia coli* are used to prevent various diseases by outcompeting pathogenic microorganisms, modifying the function of the microbial community, and regulating the immune response. In order to treat IBD, diarrhea, Crohn's disease, ulcerative colitis, and even cancer, next‐generation probiotics are made to change the composition of the gut microbiota and have context‐dependent effects determined by microbial and host variables. Efforts continue to genetically engineer probiotics to deliver diagnostic and therapeutic agents [184, 185]. Probiotics have varying impacts on gut microbiota, according to a recent meta‐analysis that highlighted conflicting results and unsolved mechanistic issues [186].

### 10.2 Subtractive Therapies

#### 10.2.1 Bacteriocins

Bacteriocins are ribosomally synthesized antimicrobial peptides that selectively inhibit pathogen growth. They are produced by most gut microbes and have functions in competitive niche colonization, protecting against pathogen colonization, and enhancing immune defenses [184, 187]. Bacteriocins are also useful for food preservation, gastrointestinal problems such as peptic ulcers, personal care products like vaginal creams and anti‐acne preparations, and maintaining oral health [187, 188]. A recent study revealed that *Pseudomonas aeruginosa's* S‐type pyocins show broad antibacterial efficacy by inhibiting both Gram‐positive ( *S. aureus* , *E. hirae* ) and Gram‐negative (*Proteus* spp.) pathogens in vitro [189]. However, the emergence of microbial resistance remains a key limitation.

#### 10.2.2 Bacteriophages

Bacteriophages attack bacteria and have been exploited to kill antibiotic‐resistant pathogens [190, 191]. They have been used to address osteomyelitis, *Pseudomonas* infections in the lungs, acne, diabetic foot ulcers, and inflammatory disorders caused by pathogenic *Escherichia coli* . Engineered phages harboring CRISPR complexes can selectively kill problematic strains. Nevertheless, recent clinical research highlighted translational issues in targeting and host–microbiome interactions by revealing varying success rates in phage therapy trials, many of which do not exhibit obvious benefit despite safety [192].

### 10.3 Modulatory Therapies

Modulatory therapies influence gut microbiota or their interactions through lifestyle and dietary changes, exercise, and medications [193, 194].

#### 10.3.1 Diet and Lifestyle

The composition of the gut microbiome is largely determined by food [195]; gluten‐free, low‐fiber, fermentable oligo‐di‐mono‐saccharide and polyol (FODMAP)‐restricted, and high‐protein diets all result in different microbial changes [196, 197]. While adequate dietary fiber maintains the integrity of the gut barrier and mucus layer, physical activity raises SCFA levels and supports microbial balance [198, 199]. Microbial diversity is affected by micronutrients like vitamin D [199, 200]. Vitamin D has a major impact on gut microbiota composition [201]. Vitamin D shows context‐dependent effects influenced by host and microbial factors. It has been demonstrated in studies that vitamin D administration changes the microbiota's makeup, particularly in illness. Vitamin D_3_ administration increases beneficial butyrate‐producing species such as *Akkermansia*, *Faecalibacterium*, and *Coprococcus* in multiple sclerosis [202], whereas vitamin D changes the microbiota in the stomach and airways in cystic fibrosis [203]. Through these effects, diet can increase beneficial metabolites, such as SCFAs, and improve the growth of protective microbial taxa. Interventions, including ketogenic diets and fatty acid based therapies, can alter microbiota in epilepsy and liver disease, with additional butyrate precursors further promoting gut health [204, 205].

#### 10.3.2 Non‐Dietary Modulators

Alcohol consumption, smoking, and exposure to antibiotics disturb the microbiome balance in the body. However, studies indicated that microbial diversity and composition can be largely restored but with variable efficacy across individuals by reducing or eliminating these variables [185]. Patients with alcohol use disorder experience improvement in gut microbiota profiles with enhanced butyrate‐producing capacity after alcohol withdrawal [206]. Smoking cessation boosts beneficial Firmicutes and Actinobacteria while decreasing *Bacteroidetes* and *Proteobacteria* [207]. Microbiome restoration techniques like commensal bacterial consortia and FMT showed stronger restoration potential than lifestyle modification alone. These interventions showed microbial balance and decrease colonization by multidrug‐resistant organisms and antibiotic resistance gene burden [208]. Antibiotic use markedly reduces microbial diversity, increases the risk of *Clostridioides difficile* infection, and leads to the development of antibiotic resistance [168].

### 10.4 Psychobiotics

Psychobiotics, such as probiotics, prebiotics, postbiotics, synbiotics, and FMT, support mental health by modulating the gut–brain axis through the vagus nerve, neuroendocrine pathways, and immune regulation. These interventions impact cognitive and emotional processes by regulating the HPA axis, neurotransmitter production, and inflammatory responses. For instance, *Lactobacillus rhamnosus* *GG* and *Bifidobacterium infantis* increase IL‐10, with associated anti‐inflammatory effects and protection against disruption of the blood–brain barrier. Certain lactobacilli produce acetylcholine, and spore‐forming bacteria promote serotonin synthesis. Psychobiotic interventions show potential as treatments for Parkinson's disease, Alzheimer's disease, Tourette syndrome, autism, insomnia, and mood disorders by reducing depression and anxiety; however, larger clinical trials are required to confirm this potential [185]. Microbiome‐targeted therapies are shown in Table 3.

**TABLE 3:** Microbiome‐based therapeutic approaches and their mechanisms of action.

Therapy type	Examples/Strategy	Mechanism of action	Applications/Targeted conditions	References
Additive therapy	Fecal microbiota transplantation (FMT)	Restores gut microbial balance by transferring healthy microbiota	Recurrent *Clostridioides difficile* infection, metabolic disorders, liver diseases, neurological disorders, adjunct in cancer therapy	[184, 185]
	Probiotics (lactobacilli, *Bifidobacteria*, engineered strains)	Compete with pathogens, modify microbial communities, regulate the immune response	Inflammatory bowel disease, diarrhea, Crohn's disease, ulcerative colitis, cancers	[184]
Subtractive therapy	Bacteriocins	Ribosomally synthesized antimicrobial peptides selectively inhibit pathogens	Gut infections, oral health, vaginal creams, anti‐acne, gastrointestinal disorders	[187, 188]
	Bacteriophages (natural or CRISPR‐engineered)	Kill targeted bacteria, including antibiotic‐resistant strains	Osteomyelitis, *Pseudomonas* infections of the lungs, acne, diabetic foot ulcers, pathogenic *Escherichia coli* infections	[185, 190]
Modulatory therapy	Diet and lifestyle	Alter microbial composition, increase short‐chain fatty acid (SCFA) production, support barrier integrity	Epilepsy, liver disease, general gut health, metabolic disorders	[193, 198]
	Non‐dietary modulators (alcohol consumption, smoking, antibiotic use)	Disrupt microbiota composition, reduce SCFAs, increase gut permeability	Crohn's disease, *C. difficile* infection, antibiotic resistance	[168, 209, 210]
Psychobiotics	Probiotics, prebiotics, postbiotics, synbiotics, FMT	Modulate the gut–brain axis via the vagus nerve and neuroendocrine and immune pathways; regulate the hypothalamic–pituitary–adrenal axis, neurotransmitters, and inflammation	Mental health, cognition, depression, anxiety, Parkinson's disease, Alzheimer's disease, Tourette syndrome, autism, insomnia	[185]

## 11 Methodological Challenges and Rigor in Microbiome Research

There are many different approaches used in microbiome research. Studies reveal that 16S rRNA sequencing frequently underrepresents taxonomic resolution and detects fewer taxa than shotgun metagenomics, which offers more species‐level detail but is more expensive and dependent on technical factors like reference databases and bioinformatics workflows [211]. Reproducibility and causal inference are frequently complicated by batch effects, uneven pipelines, and inter‐individual variability in bioinformatics methodologies. Functional interpretation of the microbiome is hindered by the limited integration of many omics, particularly metabolomics. Robust interpretation is hampered by issues such as data heterogeneity, unknown metabolites, and analytical restrictions, even though combining sequencing data with metabolomic profiling can offer deeper insights into microbial activities and host–microbe interactions [212]. Additionally, the majority of illness relationships are correlational, and the use of causal inference techniques is still in its infancy [213]. Furthermore, host genetics and individual variability play major roles in microbiome variation and affect reproducibility across cohorts [214].

## 12 Limitations and Future Perspectives

The global burden of disease study identifies the major challenges facing the health sector worldwide: obesity and malnutrition, cardiovascular disease, gastrointestinal conditions, diabetes, antimicrobial resistance, and cancers [38, 215]. The rise of COVID‐19 has brought to the forefront the need to develop a stronger defense mechanism against disease outbreaks [216]. The human microbiome is an adaptive genome with largely unexplored potential.

Preservation of microbial diversity, protection of adaptive genome components from loss, and setting up microbiome banks require a worldwide collaborative approach [217]. This might lead to increased crop productivity, improved plant resistance to climate change [218, 219], and protection for endangered species [220].

Microbiome‐targeted medicines have advanced, yet there are still significant obstacles. Population‐specific microbial signatures, poor repeatability across cohorts, and a lack of integration between host genetics and multi‐omics data make it difficult to customize medication. Research gaps also include the lack of investigation of specific tissue site responses; the role of non‐bacterial organisms, including viruses, fungi, and archaea; extra‐gut microbiomes; inter‐site communication (gut–lung, gut–skin, and gut–oral); and how microbes within tumors drive mutation, inflammation, immune evasion, and metastasis. Targeting these interactions could enhance cancer therapy [38]. Studies of populations with rare diseases, those undergoing surgery, and transplant patients are generally lacking. Transforming microbiome interventions into dependable, precise treatments will require addressing these problems.

Microbiomes play key roles in disease diagnosis, classification, treatment, and prognosis. Adaptive genomic factors influence diet and medication responses. Application of the idea of “acquired microbial immunity” may help reduce allergies, autoimmune problems, and intestinal infections, although its potential risks require careful evaluation. Standardized guidelines, together with specialized microbial clinical units, might favor its safe translation into clinical practice [38].

Newly emerging microbiome‐targeting interventions, such as prebiotics, probiotics, synbiotics, and postbiotics, have been found to modulate the gut microbiome and the intestinal barrier by mechanisms such as microbiome enrichment, the generation of SCFAs, reinforcement of the intestinal barrier, and immune modulation. Despite the inconsistent effectiveness of these biotic approaches, their targeted application has been found to be beneficial for the promotion of personalized nutrition and the development of adjunct therapies [221].

Novel approaches, such as CRISPR‐based phage therapy, microbially produced bioactive chemicals, designed probiotics, and microbiome engineering technologies, offer encouraging paths to get past present obstacles. Biome engineering provides routes to direct the therapeutic microbiome. This includes engineered bacteria such as *Escherichia coli* Nissle 1917 and SYNB1020 to counter hyperammonemia, microbially produced compounds such as β‐carotene and violacein, phage approaches using CRISPR against *Clostridioides difficile*, CRISPR‐driven targeted strain modification, gene clusters, and even swarmbots [38, 222]. Although there is still a lack of clinical validation and long‐term safety evidence, these approaches support personalized microbiome treatment by customizing interventions to host genetics, microbial composition, and illness context.

The majority of the studies presented here are from high‐income nations [38], indicating that international associations and publishing organizations need to be more equitable. For the sake of human health, the microbiome's potential must be realized. Future space colonization may pose health problems if the innate genome is the main emphasis and the adaptable genome is neglected. Thus, an interplanetary microbiome project would be required.

## 13 Conclusion

In conclusion, the human microbiome is an integrated, highly dynamic ecosystem that is critical to both the health and disease across all organ systems. Results from clinical and experimental studies demonstrate that host physiology, environmental factors, and lifestyle choices impact microbial communities, and that dysbiosis is associated with immunological dysregulation, metabolic disorders, and the progress of chronic diseases. Microbiome‐targeted nutritional strategies, probiotics, bacteriophages, FMT, and personalized microbiome‐based strategies for disease prevention and clinical management are a few of the exciting therapeutic options made possible by advances in multi‐omics technologies and microbiome research. However, significant obstacles still exist, especially in the areas of clinical adoption, standardization, and determination of causality. To fully apply safe, accurate, and customized microbiome‐based approaches for clinical care and disease prevention, more integrative and long‐term research is necessary.

## Funding

The author has nothing to report.

## Ethics Statement

The author has nothing to report.

## Consent

The author has nothing to report.

## Conflicts of Interest

The author declares no conflicts of interest.

## Data Availability

The author has nothing to report.
